# The pan genome analysis of *WOX* gene family in apple and the two sides of *MdWUS-1* in promoting leaf-borne shoot

**DOI:** 10.1093/hr/uhaf117

**Published:** 2025-07-11

**Authors:** Lin Liu, Yafei Shu, Yue Wang, Mingyue Liu, Shuxin Xu, Xiaofan Lu, Yu Zhang, Luyao Yu, Ze Tao, Jiale Wang, Bingkun Ge, Pengzhen Cui, Changai Wu, Jinguang Huang, Kang Yan, Chengchao Zheng, Guodong Yang, Xin Tian, Shizhong Zhang

**Affiliations:** College of Life Sciences, Shandong Agricultural University, Tai’an 271018, China; College of Life Sciences, Shandong Agricultural University, Tai’an 271018, China; College of Life Sciences, Shandong Agricultural University, Tai’an 271018, China; College of Life Sciences, Shandong Agricultural University, Tai’an 271018, China; College of Life Sciences, Shandong Agricultural University, Tai’an 271018, China; College of Life Sciences, Shandong Agricultural University, Tai’an 271018, China; College of Life Sciences, Shandong Agricultural University, Tai’an 271018, China; College of Life Sciences, Shandong University, Qingdao 266237, China; College of Horticulture, Shenyang Agricultural University, Shenyang 110866, China; College of Horticulture, Shenyang Agricultural University, Shenyang 110866, China; Biotechnology Research Institute, Chinese Academy of Agricultural Sciences, Beijing 100081, China; College of Food Science and Engineering, Shandong Agricultural University, Tai’an 271018, China; College of Life Sciences, Shandong Agricultural University, Tai’an 271018, China; College of Life Sciences, Shandong Agricultural University, Tai’an 271018, China; College of Life Sciences, Shandong Agricultural University, Tai’an 271018, China; College of Life Sciences, Shandong Agricultural University, Tai’an 271018, China; College of Life Sciences, Shandong Agricultural University, Tai’an 271018, China; College of Life Sciences, Shandong Agricultural University, Tai’an 271018, China; College of Life Sciences, Shandong Agricultural University, Tai’an 271018, China

## Abstract

Unlike animals, plants are sessile organisms that cannot move freely in response to fluctuating and complex environments. As a result, plant development follows post-embryonic processes, enabling flexible developmental strategies to adapt to changing environment. The *WUSCHEL-*related homeobox (*WOX*) gene family plays a crucial role in regulating these post-embryonic processes in plants. In this study, we performed an evolutionary analysis of the *WOX* gene family across 29 plant species, isolating a total of 330 *WOX* family genes. Our study identified a fern protein with similar length and conserved motifs to WUS gene of spermatophyte, suggesting that the modern clade of the *WOX* family may have already diverged in ferns. Furthermore, we conducted a pan-genome analysis of the *WOX* family in *Malus*, examining the number and gene characteristics of *WOX* family members across eight varieties. The promoter elements of *WUS-1*, *WUS-2*, *WOX5-1*, and *WOX5-2* in different *Malus* varieties were analyzed further. Additionally, we examined the expression patterns of modern clade *WOX* family members in developing tissues and during leaf-borne shoot regeneration of *Malus*. We developed the transgenic lines with inducible overexpression of *MdWUS-1* or *MdWOX5-1*, which revealed that mild upregulation of *MdWUS-1* significantly promoted leaf-borne shoot formation, while strong upregulation of *MdWUS-1* led to browning and death of explants, likely due to oxidative stress. These findings provide new insights into the evolution of the *WOX* gene family from ferns into seed plants and lay the foundation for further studies on the spatiotemporal regulation of gene expression during shoot regeneration.

## Introduction

Due to the sessile nature of most terrestrial plants and the complexity and variability of their environments, plants have evolved more flexible developmental strategies compared to animals. During embryonic development, plants generate only a few primordial tissues and organs. Most organs, such as lateral roots, stems, true leaves, and flowers, arise from the apical meristems after seed germination [1]. As a result, the apical meristem plays a crucial role in plant development [2]. The apical meristem contains a cluster of stem cells, serving as a reservoir for post-embryonic organogenesis. [3]. Central to the regulation of stem cell maintenance in the apical meristems are key factors such as WUSCHEL (WUS) in shoot and WOX5 in root [4, 5]. These genes are crucial for maintaining the balance between stem cell self-renewal and differentiation, ensuring the continuous growth and development of plant tissues throughout the plant’s life cycle.

*WUS* and *WOX5* belong to the modern subclade of the *WOX* family, which is divided into three clades: the ancient clade, the intermediate clade, and the modern clade. These clades likely reflect an evolutionary progression from lower to higher organisms [6]. The ancient clade originated in *Ostreococcus lucimarinus*, the intermediate clade emerged in ferns, and the modern clade including WUS and WOX5 differentiated in gymnosperms. In Arabidopsis, the modern clade is represented by the WUS gene, which is why this clade is often referred to as the WUS clade [7]. Both WUS and WOX5 contain three conserved motifs, homeodomain (HD) motif to bind DNA, WUS-box motif to regulate the shoot stem-cell population and the floral pattern, and ERF-associated amphiphilic repression (EAR) motif involved in transcriptional repression.

Recent studies have identified a gene in *Ceratopteris richardii* that belongs to the modern clade and was named *WUSCHEL-like* (*WUL*). However, due to the large molecular weight of the WUL protein, it was unable to perform the typical function of the WUS gene in maintaining the shoot apical meristem (SAM). Only a truncated version of WUL exhibited functional activity [8]. This finding suggests that the ancestor of the *WUS* gene had already emerged in ferns, even though a *WUS* gene with a length comparable to those found in modern lineages has not been identified in ferns to date.

Biological evolution is widely recognized as a gradual process [9]. It remains unclear whether ferns have evolved a typical *WUS* gene comparable in length to that of the spermatophyte. The completion of genome sequencing for several true ferns, including *Ceratopteris richardii [**10**]*, *Adiantum_capillus-veneris* [11], *Salvinia cucullata*, *Azolla filiculoides* [12], and *Alsophila spinulosa* [13] offers new opportunities to explore the evolutionary origins of the modern clade within these taxa. Furthermore, pan-genome sequencing establishes a foundation for exploring the relationship between the gene structure and functional diversity of WUS and WOX5 across different varieties within *Malus*.

*WUS* and *WOX5* are key regulators expressed in the organizing center (OC) of SAM and the quiescent center (QC) of root apical meristem (RAM), respectively, where they play pivotal roles in stem cell maintenance and organogenesis, including lateral root formation, shoot differentiation, and *de novo* shoot formation [14–16]. Generally, elevated expression of these two genes enhances regeneration [15, 17, 18]. However, recent studies have uncovered a more intricate role of *WUS* in organogenesis, demonstrating that increased expression does not always correlate with enhanced regeneration. For instance, strong upregulation of *ZmWUS2* has been found to be less effective in promoting *de novo* meristem induction in tobacco and leaf-based regeneration in monocots compared to mild upregulation [19, 20]. Sustained overexpression of *ZmWUS2* driven by a strong callus-specific promoter often leads to necrosis of the callus, resulting in regeneration of only non-transgenic plants [21]. Our previous study found that a 10-fold induction of *MdWUS-1* enhanced the efficiency of both root-borne shoot (RBS) and leaf-borne shoot (LBS) regeneration. However, a 700-fold induction of *MdWUS-1* led to growth cessation and explant death. It is an interesting question that why excessively high levels of *WUS* gene expression causes callus, root, and leaf browning and death.

## Results

### Evolutionary relationship of *WOX* family in plants

The *WOX* gene family belongs to homeodomain superfamily [22]. To investigate the evolutionary history of the *WOX* gene family, we selected 29 plant genomes representing a broad range of plant lineages, arranged in order of evolutionary complexity from lower to higher plants. These genomes were then used to construct a phylogenetic tree of homeodomain-containing proteins (Fig. S1A).

Given the critical role of ferns in the evolution of modern clade, we included all the ferns whose genomes had been sequenced for detailed analysis. The species selected for this study include one protist (*Porphyra umbilicalis, P. umbilicalis*), four algae (*Chlamydomonas reinhardtii*, *C. reinhardtii*; *Dunaliella salina*, *D*. *salina*; *Botryococcus braunii*, *B*. *braunii*; *Ostreococcus lucimarinus*, *O*. *lucimarinus*), two bryophyta (*Marchantia polymorpha*, *M*. *polymorpha*; *Physcomitrium patens*, *P*. *patens*), eight pteridophyta (*Diphasiastrum complanatum*, *D*. *complanatum*; *Selaginella moellendorffi*, *S*. *moellendorffi*; *Salvinia cucullata*, *S cucullata*; *Ceratopteris richardii*, *C*. *richardii*; *Adiantum_capillus-veneris*, *A*. *capillus-veneris*; *Azolla filiculoides*, *A*. *filiculoides*; *Alsophila spinulosa*, *A*. *spinulosa*; *Marsilea vestita*, *M*. *vestita*), one gymnosperms (*Thuja plicata*, *T*. *plicata*), seven eudicots (*Arabidopsis thaliana*, A. *thaliana*; *Solanum lycopersicum*, S. *lycopersicum*; *Glycine max*, G. *max*; *Populus trichocarpa*, P *trichocarpa*; *Malus domestica*, M. *domestica*; *Prunus persica, P*. *persica, Cucumis sativus*, *C*. *sativus*), as well as six monocots (*Cocos nucifera*, C. *nucifera*; *Hordeum vulgare*, H. *vulgare*; *Triticum aestivum*, T. *aestivum*; Osativa Kitaake, O. Kitaake; *Setaria italica*, S. *italica*; *Zea mays*, Z. *mays*). In the phylogenetic tree, the *WOX* family naturally clusters together, encompassing 25 species out of 29 species examined (Fig. S1A).

The phylogenetic analysis identified a total of 330 *WOX* genes across the 29 selected species. Consistent with previous studies, the first *WOX* gene emerged in the unicellular green alga *O*. *lucimarinus*, a late-diverging lineage within algal and an early-diverging lineage of green plants at the same time [6]. As plant species evolved, the *WOX* gene family underwent diversification, forming three distinct clades: the ancient clade (80 members), intermediate clade (97 members), and modern clade (153 members) (Table 1).

**Table 1:** Gene numbers of three clades of *WOX* gene family of all species

Category	Species	Gene number				
		*Homeobox* family	*WOX* family	Ancient clade	Intermediate clade	Modern clade
Protist	*Porphyra umbilicalis*	4	0	0	0	0
Algae	*Dunaliella salina*	3	0	0	0	0
	*Botryococcus braunii*	5	0	0	0	0
	*Chlamydomonas reinhardtii*	5	0	0	0	0
	*Ostreococcus lucimarinus*	6	1	1	0	0
Bryophyta	*Marchantia polymorpha*	17	1	1	0	0
	*Physcomitrella patens*	58	3	3	0	0
Fern	*Marsilea vestita*	55	3	3	0	0
	*Diphasiastrum complanatum*	79	7	7	0	0
	*Selaginella moellendorffii*	33	8	7	1	0
	*Salvinia cucullata*	48	5	4	1	0
	*Adiantum capillus-veneris*	64	8	3	4	1
	*Alsophila spinulosa*	110	14	9	4	1
	*Ceratopteris richardii*	88	25	3	20	2
	*Azolla filiculoides*	61	8	2	4	2
Gymnosperms	*Thuja plicata*	89	24	4	2	18
Monocots	*Oryza sativa*	92	13	1	6	6
	*Setaria italica*	95	13	1	5	7
	*Hordeum vulgare*	94	14	1	5	8
	*Triticum aestivum*	192	25	7	6	12
	*Zea mays*	139	24	2	9	13
	*Cocos nucifera*	353	18	2	5	11
Eudicots	*Prunus persica*	75	10	2	2	6
	*Cucumis sativus*	81	11	2	2	7
	*Arabidopsis thaliana*	97	16	3	5	8
	*Solanum lycopersicum*	112	11	1	2	8
	*Populus trichocarpa*	134	18	3	4	11
	*Malus domestica*	140	18	3	4	11
	*Glycine max*	228	33	6	6	21
	Total	2556	330	80	97	153

**Figure 1:**
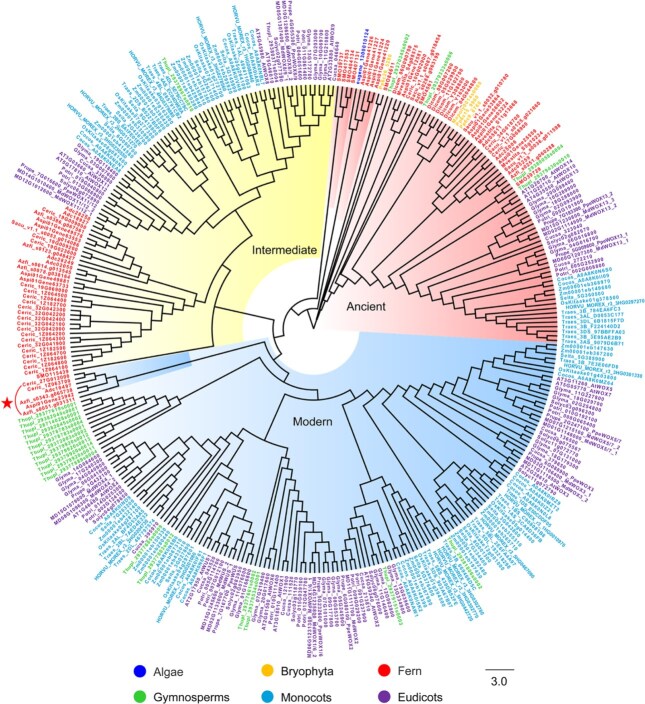
Phylogenetic relationships of *WOX* family genes. The background colors of the phylogenetic tree represent the three subclades of the *WOX* gene family: the pink region corresponds to the ancient subclade, the yellow region to the intermediate subclade, and the blue region to the modern subclade. The labels surrounding the phylogenetic tree indicate different species. Labels starting with ‘eu’ represents *Ostreococcus lucimarinus*, ‘Ma’ represents *Marchantia polymorpha*, ‘Pp’ represents *Physcomitrium patens*, ‘Di’ represents *Diphasiastrum complanatum*, ‘SM’ represents *Selaginella moellendorffi*, ‘Ce’ represents *Ceratopteris richardii*, ‘Az’ represents *Azolla filiculoides*, ‘As’ represents *Alsophila spinulosa*, ‘Ad’ represents *Adiantum capillus*, ‘Sa’ represents *Salvinia cucullata*, ‘Mv’ represents *Marsilea vestita*, ‘Th’ represents *Thuja plicata*, ‘AT’ represents *Arabidopsis thaliana*, ‘So’ represents *Solanum lycopersicum*, ‘Gl’ represents *Glycine max*, ‘Po’ represents *Populus trichocarpa*, ‘MD’ represents *Malus domestica*, ‘Pr’ represents *Prunus persica*, ‘Co’ represents *Cocos nucifera*, ‘HO’ represents *Hordeum vulgare*, ‘Tr’ represents *Triticum aestivum*, ‘Os’ representing *Osativa Kitaake*, ‘Se’ representing *Setaria italica*, ‘Zm’ representing *Zea mays*. The members enclosed by the arc, which marked with a star, represent fern genes classified into the modern clade.

In the current phylogenetic framework, the ancient clade spans from unicellular green algae (*O. lucimarinus*) to seed plants, while the intermediate clade appeared from true ferns to seed plants. The fern *C. richardii* contains as many as 20 members in the intermediate clade, whereas the *Zea mays*, a seed plant, has only 9. Notably, in the *WOX* family, a small group of fern genes was found to cluster in the modern clade, comprising six members. These include one member from *A. capillus-veneris*, one from *A. spinulosa*, two from *C. richardii*, and two from *A. filiculoides* (Fig. 1). This clustering suggests that true ferns, represented by *C. richardii*, may serve as transitional species in the evolutionary shift of the *WOX* family from the ancient clade to the modern clade.

### A typical *WUS* gene of modern clade exists in the fern plant *a. filiculoides*

**Figure 2:**
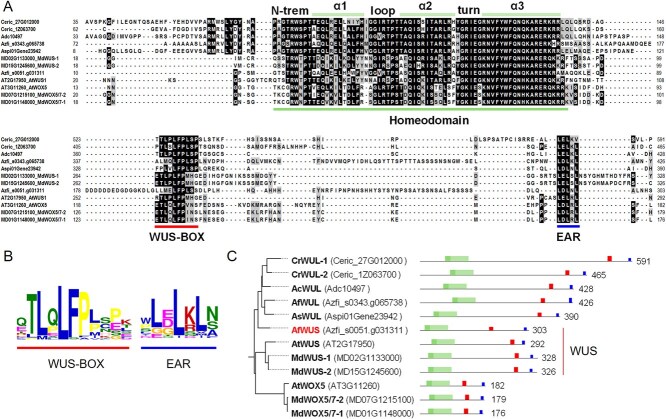
The Phylogenetic relationships of *WUS* gene in fern and seed plants. (A) Sequence alignment of *WUS* and *WOX5* genes from all modern clade members of ferns, *Malus*, and Arabidopsis. For clarity, the sequences are truncated, with the numbers at both ends representing the actual sequence length. (B) Protein logos of the WUS–BOX and EAR domains for all species. (C) Phylogenetic relationships, protein lengths, and conserved domains of the *WUS* and *WOX5* genes from all modern clade members of ferns, *Malus*, and Arabidopsis.

To further investigate the evolutionary relationship of these six fern *WOX* genes within the modern clade, we performed a conserved domain analysis. Our results revealed that these six genes share three conserved domains similar to the *WUS* and *WOX5* genes in angiosperm (Fig. S1B). Sequence alignment confirmed that all six genes contain the characteristic domains of WUS and WOX5: the Homeodomain, WUS-BOX, and EAR domain (Fig. 2A). The WUS-BOX sequence is characterized by the ‘LFP’ motif, while the EAR domain contains the sequence motif ‘LXLXLX’ (Fig. 2B).

Among these six members, *Ceric 27G012000* corresponds to the previously reported *CrWUL* in *Ceratopteris richardii* [8]. Additionally, we identified another homologous gene in *C. richardii*, *Ceric 1Z063700*, which we have named *CrWUL2*. The genes *Adc10497* from *A. capillus-veneris (AcWUL)*, *Aspi01Gene23942* from *A. spinulosa (AsWUL)*, and *Azfi_s0343.g065738* from *A. filiculoides* (*AfWUL*) were also identified. All these members consist of over 390 amino acids, which is considerably longer than the WUS proteins from Arabidopsis and *Malus*, which range from 292 to 328 amino acids.

Interestingly, another member from *A. filiculoides*, *Azfi_s0051.g031311*, has a protein length of 303 amino acids, which falls between the lengths of WUS proteins from Arabidopsis and *Malus*. Based on evolutionary relationships, *Azfi_s0051.g031311* was positioned between the ferns and angiosperms gene clades. Considering its sequence length, the presence of all conserved domains, and its evolutionary positioning, we have named this gene *AfWUS* (Fig. 2C). These findings indicated that the typical *WUS* gene did not evolve only after the emergence of seed plants but likely existed in ferns already. This provides new insights into the evolutionary history and functional role of *WUS* in plant developmental processes, highlighting its early presence in the plant lineage and its potential role in regulating development in both ferns and seed plants.

### The pan-genome analysis of the *WOX* family of *Malus*

**Figure 3:**
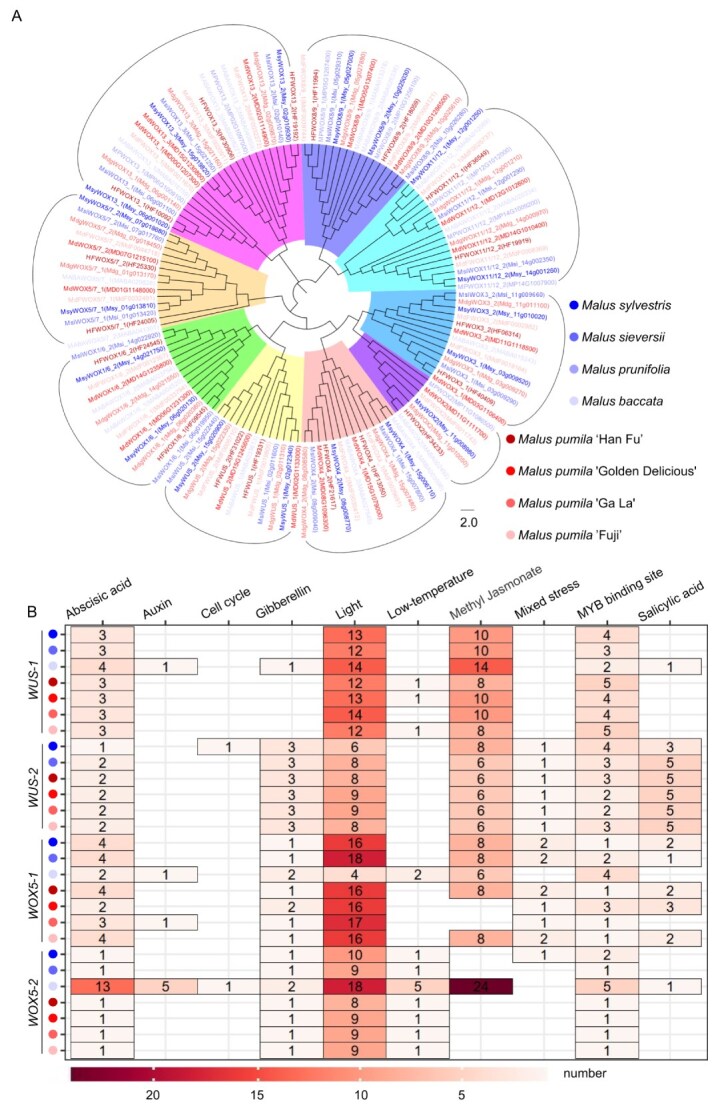
The pan-genome analysis of the *WOX* family in *Malus*. (A) Phylogenetic relationships of *WOX* family proteins of eight varieties of *Malus*. The background colors of the phylogenetic tree represent the members of the *WOX* gene family of all eight varieties. (B) The promoter *cis*-elements analysis for *WUS-1*, *WUS-2*, *WOX5-1*, and *WOX5-2* across seven cultivars of *Malus*. The colors of the dots on the left represent different varieties. The number in boxes represents the number of responding elements.

Deciduous fruit trees, such as *Malus* trees, are often subjected to complex and variable environmental conditions, which may drive the evolution of more sophisticated developmental strategies. We conducted a comprehensive analysis of the *WOX* gene family in eight *Malus* varieties, including four wild varieties (*Malus sylvestris*, *Malus sieversii*, *Malus prunifolia*, and *Malus baccata*) and four cultivated varieties (‘Han Fu’, ‘Golden Delicious’, ‘Ga La’, and ‘Fuji’). Phylogenetic analysis revealed that all cultivated varieties contain 18 *WOX* family members, including 11 members in the modern branch, 4 members in the intermediate branch, and 3 members in the ancient branch. In contrast, the number of *WOX* family members in wild species varied: *Malus sylvestris* had 18 members, *Malus sieversii* had 17 members, *Malus prunifolia* had 8 members, and *Malus baccata* had 16 members (Fig. 3A).

Comparative synteny analysis across all eight varieties showed that strong collinearity among all *WOX* members, with no chromosomal rearrangements observed between varieties (Fig. S2). Gene structure analysis of the *WOX* family members revealed that the coding sequence regions of *WUS-1* and *WUS-2* were identical in terms of position and length across all varieties, indicating a high degree of conservation of these genes (Fig. S3). Furthermore, the amino acid sequence alignment of *WOX* family members revealed that most pairs of homologous genes exhibited segmental or individual amino acid insertions or deletions. Additionally, considerable variations in sequence length, including insertions or deletions, were observed within the same gene across different varieties. Notably, the amino acid sequences of *WUS-1* and *WUS-2* were highly conserved across *Malus* varieties, with only a few single amino acid substitutions detected (Fig. S4A–J).

Promoter regions play a decisive role in regulating the transcriptional activity of genes. To better understand the regulatory mechanism underlying the expression of *WOX* genes, we performed a detailed *cis*-element analysis in the promoter regions of *WUS-1*, *WUS-2*, *WOX5-1*, and *WOX5-2* across different *Malus* cultivars (Fig. 3B, Table S4). *Malus prunifolia* was excluded from this analysis due to its possession of only eight *WOX* family members. Our analysis revealed the presence of several *cis*-elements responsive to various hormones and environmental cues, including abscisic acid, auxin, cell cycle, gibberellin, light, low temperature, methyl jasmonate, mixed stress, salicylic acid and MYB binding sites. Intriguingly, we observed that all seven cultivars contained a high number of light-responsive elements in the promoters of *WUS-1*, *WUS-2*, *WOX5-1*, and *WOX5-2*, with *WUS-1* and *WOX5-1* generally containing more light-responsive elements than *WUS-2* and *WOX5-2*. The *WUS-2* promoter was particularly enriched with salicylic acid-responsive elements compared to the other three genes. Both *WUS-1* and *WUS-2* promoters contained multiple methyl jasmonate-responsive elements, while the promoters of *WOX5-1* in ‘Golden Delicious’ and ‘Ga La’ lacked methyl jasmonate-responsive elements. Interestingly, the *Malus baccata* was the only one cultivar whose *WOX5-2* promoter harbors a significant number of methyl jasmonate-responsive elements. Overall, *WUS-1* and *WUS-2* promoters were characterized by a higher number of methyl jasmonate-responsive elements than those of *WOX5-1* and *WOX5-2.* Notably, the promoter of *WOX5-2* in *Malus baccata* contained a greater number of all identified c*is*-elements compared to other cultivars, suggesting that the expression pattern of *WOX5-2* in *Malus baccata* may be more distinctive compared to other cultivars.

### The expression patterns of modern clade *WOX* genes in developing tissues and process of LBS regeneration in *Malus*

**Figure 4:**
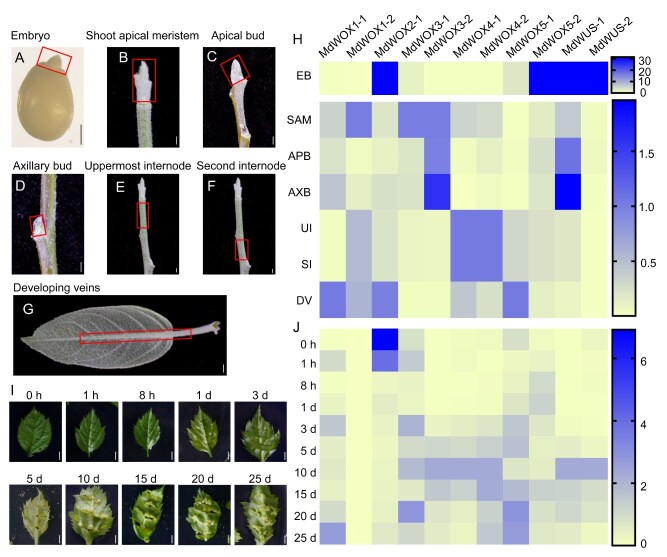
Tissue expression patterns and expression changes during the LBS process in the modern clade of the *WOX* gene family in *Malus*. (A-G) Tissues sampled for quantitative expression analysis. (H) Relative expression levels of 11 modern clade *WOX* genes across the seven tissues. ‘EB’ represents embryos, ‘SAM’ represents shoot apical meristems, ‘APB’ represents apical buds, ‘AXB’ represents axillary buds, ‘UI’ represents uppermost internode, ‘SI’ represents second internode, ‘DV’ represents developing leaves. (I) Leaf states at 10 sampling time points during the LBS process, where ‘h’ represents hours and ‘d’ represents days. (J) Relative expression levels of 11 modern clade *WOX* genes across the 10 LBS time points. The bars in this figure are 0.2 cm.

To explore the impact of modern clade members of *MdWOX* family on plant development, we conducted a detailed analysis of their expression patterns in seven regeneration competent tissues, including embryo, SAM, apical bud, axillary bud, uppermost internode, second internode, and developing veins (Fig. 4A–G). Quantitative expression analysis revealed distinct expression patterns across the tested tissues. *MdWUS1*, *MdWUS2*, *MdWOX5-2*, and *MdWOX2-1* genes were highly expressed in the embryo, consisting with their critical role in embryo development. High expression levels of *MdWOX1-2*, *MdWOX3-1*, and *MdWOX3-2* were observed in the actively growing SAM. In the apical buds and axillary buds, *MdWOX3-2* and *MdWUS-1* exhibited higher expression levels, with axillary buds showing greater expression than apical buds. The expression patterns of all modern clade members of *MdWOX* family were identical between the first and second internodes, indicating a uniform expression during stem elongation. In developing leaf veins, *MdWOX1-1*, *MdWOX2-1*, and *MdWOX5-1* were expressed at relatively high levels (Fig. 4H).

We further analyzed the temporal expression patterns of *MdWUS* family genes throughout the LBS regeneration process in *Malus* (Figs 4I and S5A–G). During this process, most of the genes exhibited an initial increase in expression levels followed by a subsequent decrease, with the exception of *MdWOX2-1* showing a continuous decline in expression throughout the process. Notably, the expression levels of both *MdWUS-1* and *MdWUS-2* peaked on day 10, after which their expression steadily decreased between days 15 and 25. This pattern was well aligned with the phenotypic observations, which revealed that the first 10 days were characterized predominantly by callus growth, after which bud initiation started to emerge. The synchronized peak expression of both *MdWUS-1* and *MdWUS-2* on day 10 followed by their decline as bud initiation took place, suggests their potential role in regulating the transition from callus formation to bud development (Fig. 4T). Given the high expression levels of *MdWUS-1* in the embryo, apical buds, and axillary buds, we further performed functional analysis of *MdWUS-1* on plant development and regeneration.

### *MdWUS-1* displays a stronger function in promoting the differentiation of shoot compared to *MdWOX5-1*

**Figure 5:**
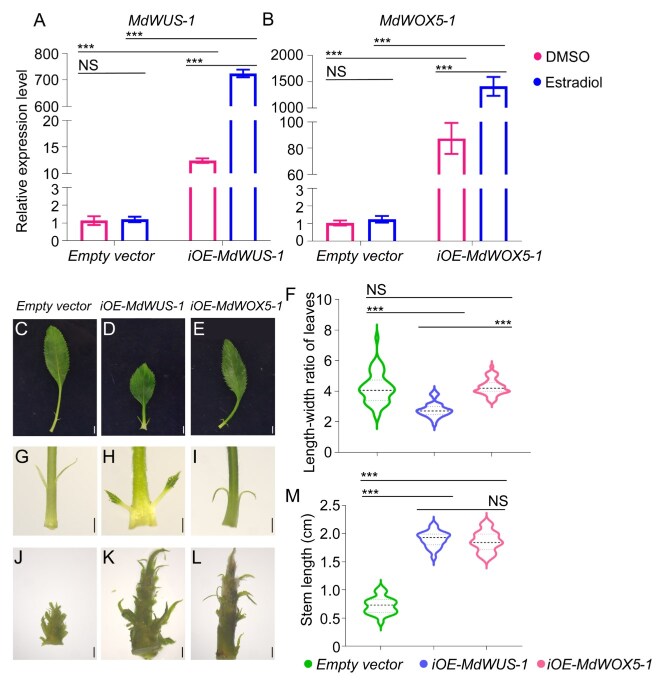
Developmental phenotypes of *iOE-MdWUS-1* and *iOE-MdWOX5-1***.** (A) Expression levels of *MdWUS-1* in transgenic lines (*iOE-MdWUS-1*) of *Malus* under the control of estradiol-inducible promoter. (B) Expression levels of *MdWOX5-1* in transgenic lines (*iOE-MdWOX5-1*) of *Malus* under the control of estradiol-inducible promoter. (C–E) Leaf phenotypes of control lines (empty vector), *MdWUS-1* transgenic lines, and *MdWOX5-1* transgenic lines. (F) Statistical analysis of leaf length-to-width ratios. (G–I) Stipule phenotypes. (J–L) Stem segment phenotypes. (M) Statistical analysis of stem segment lengths. All phenotypic observations were conducted on *in vitro*-grown seedlings without estradiol induction. The bars in this figure are 0.2 cm. Data are means ± SE (*n* = 3). Asterisks indicate significant differences by Student’s *t*-test (^*^P < 0.05).

To investigate the role of *MdWUS-1* in development and regeneration, we generated the transgenic apple plants with estradiol-inducible overexpression of *MdWUS-1*, referred to as *iOE-MdWUS-1*. Additionally, we included a transgenic line with estradiol-inducible overexpression of *MdWOX5-1* (*iOE-MdWOX5-1*) from our previous study, which showed that *MdWOX5-1* significantly promotes RBS [23]. Studies in Arabidopsis have demonstrated that mis-expressing *AtWOX5*, which is normally expressed in the QC of RAM, in the OC of SAM can functionally replace *AtWUS* [3]. This prompted us to speculate whether overexpression of these two Malus genes would result in similar phenotypes. Expression analysis revealed that, in the absence of estradiol, the transgenic lines showed moderate increases in the expression of transgenes, approximately 10–100 times higher than the control plants carrying the empty vector construct. Upon estradiol treatment, the expression level of *MdWUS-1* increased by 700-fold, while the expression level of *MdWOX5-1* increased over 1000-fold. For the control plants carrying the empty vector, estradiol had no effect on expression of *MdWUS-1* and *MdWOX5-1* (Fig. 5A and B). This result confirmed that the estradiol inducible system is effective in *Malus*, enabling precise control over the expression of the genes of interest.

Under the estradiol-free condition, both *iOE-MdWUS-1* and *iOE-MdWOX5-1* plantlets grew taller than the control plantlets. However, upon estradiol application, the *iOE-MdWUS-1* plants exhibited a striking growth cessation phenotype (Fig. S6A–F). Therefore, we analyzed the phenotypes of *iOE-MdWUS-1* and *iOE-MdWOX5-1* lines without estradiol treatment, representing a moderate upregulation scenario. First, we assessed leaf morphology by measuring the length-to-width ratio. Only *iOE-MdWUS-1* line showed a significant reduction in this ratio, resulting in a shorter leaf (Fig. 5C–F). Next, we examined stipule morphology. In control plants, the stipules were linear at the base of the leaves. Overexpression of *MdWOX5-1* resulted in curved stipules. In contrast, *MdWUS-1* overexpression caused the stipules to differentiate into leaf-like structures, suggesting that *MdWUS-1* plays a role in promoting leaf morphogenesis (Fig. 5G–I). Finally, we noticed that stems of both *iOE-MdWUS-1* and *iOE-MdWOX5-1* lines were higher than of the control plants. Interestingly, the axillary buds of the control and *iOE-MdWOX5-1* plants showed minimal elongation. In contrast, the basal axillary buds of the *iOE-MdWUS-1* lines exhibited substantial elongation, though these elongated buds did not develop into lateral branches as no mature leaves formed on the elongated buds (Fig. 5J–M). Collectively, these results regarding leaf morphology, stipule morphology, and lateral bud development indicate that the *MdWUS-1* had a more pronounced influence on shoot differentiation compared to *MdWOX5-1*. This raises the important question of whether *MdWUS-1* also plays a more significant role during bud regeneration from leaves.

### *MdWUS-1* exhibits a stronger promotive effect on LBS compared to *MdWOX5-1*.

**Figure 6:**
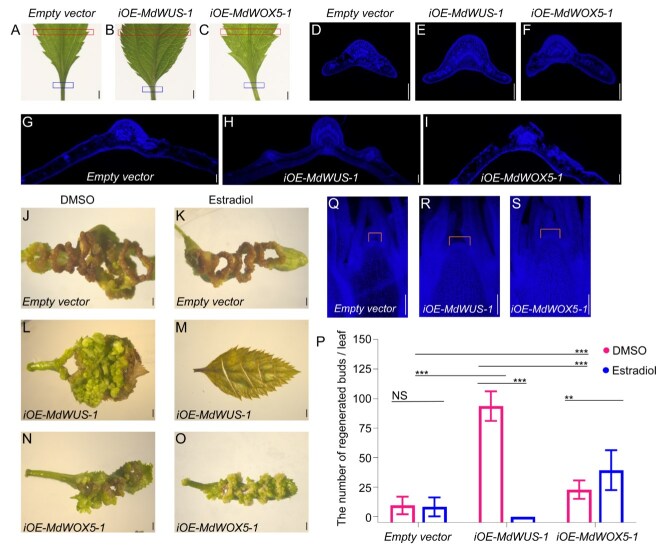
Sectional Observation and Regeneration Phenotypes of *iOE-MdWUS-1* and *iOE-MdWOX5-1*. (A–C) Morphology of leaf veins observed directly under a stereomicroscope. (D–F) Transverse sections of the leaf veins in the down-boxed areas from panels A–C. (G–I) Transverse sections of the leaf blades in the up-boxed areas from panels A–C. (J–O) LBS induction experiments: (J, L, N) without estradiol induction; (K, M, O) with 50 μM estradiol induction. (P) Statistical analysis of shoot formation per leaf in the LBS process. (Q–S) Sections of the SAM. The bars in (A–C, J–S) of this figure are 200 μm. The bars in (D–I) of this figure are 20 μm. Data are means ± SE (*n* = 3). Asterisks indicate significant differences by Student’s *t*-test (^*^P < 0.05).

Leaves are commonly used as explants for bud regeneration in *Malus*. Considering that bud regeneration tends from the leaf vein during the LBS process, we examined the vascular tissue morphology of the leaves and found that the petioles and veins of the *iOE-MdWUS-1* lines were notably thicker compared to those in the control plants (Fig. 6A–C). Histological analysis of petiole cross-sections revealed a higher proportion of small-volume cells in both *iOE-MdWUS-1* and *iOE-MdWOX5-1* lines relative to the control plants. Furthermore, the *iOE-MdWUS-1* lines exhibited a significant increase in the number of cell layers within the petioles (Fig. 6D–F). Additionally, the cell layers of adaxial surface of main veins in the *iOE-MdWUS-1* lines were more prominent than those in the *iOE-MdWOX5-1* lines and the control plants. The lateral veins in the *iOE-MdWUS-1* lines were also more elevated above the leaf surface, in contrast to the relatively flat or only slightly raised lateral veins in the *iOE-MdWOX5-1* and the control leaves (Fig. 6G–I). The regeneration process in plants originates from the xylem pole pericycle cells [24]. The increased cell layers of adaxial surface of main veins exhibit positional similarity to the xylem pole pericycle cells in roots. Therefore, the increase in these cells may be associated with the regeneration capacity of LBS.

To assess the impact of different levels of *MdWUS-1* and *MdWOX5-1* expression on LBS, we conducted LBS experiments using older leaves from control, *iOE-MdWUS-1*, and *iOE-MdWOX5-1* lines, with or without estradiol induction. Given the LBS capacity decreases with leaf age, older leaves (90 days grown in tissue culture) were selected for these experiments as they better reflect regenerative ability. The results showed that, in older leaves, the control line produced 10–20 bud points per leaf, with non-regenerating callus regions showing severe browning. The addition of estradiol had no effect on bud formation in the control line. By contrast, the *iOE-MdWOX5-1* lines exhibited a higher bud regeneration rate than the control even without estradiol induction, although some browning of the callus still occurred. Estradiol further enhanced the bud regeneration rate of *iOE-MdWOX5-1* lines, with the most notable effect being the preservation of the leaf's green color, with almost no browning observed. Strikingly, the *iOE-MdWUS-1* lines exhibited the highest bud regeneration rate under estradiol-free condition, with over 100 bud points per leaf, though callus regions without bud formation still showed browning. However, upon estradiol application, the leaves of the *iOE-MdWUS-1* lines ceased differentiation, rapidly yellowed, and eventually browned with no bud points formed at all (Figs 6J–O and S7A–F). The regeneration efficiency ranked as follows: *iOE-MdWUS-1* (DMSO) > *iOE-MdWOX5-1* (estradiol) > *iOE-MdWOX5-1* (DMSO) > control (estradiol/DMSO) > *iOE-MdWUS-1* (estradiol) (Fig. 6P).

Given the link between meristem size and regeneration efficiency, we analyzed the meristematic tissue in stem. Sectioning and staining revealed that the SAM in the *iOE-MdWUS-1* and *iOE-MdWOX5-1* lines were larger than those in the control plants. Additionally, under the same section thickness, cell density appeared higher in *iOE-MdWUS-1* and *iOE-MdWOX5-1* lines, suggesting increased cell division activity (Fig. 6Q–S). Collectively, these results indicate that moderate upregulation of *MdWUS-1* promotes the development of leaf vascular tissue and significantly boosts the LBS capacity of *Malus* leaves, while excessive *MdWUS-1* expression induces cell death and impairs regeneration.

**Figure 7:**
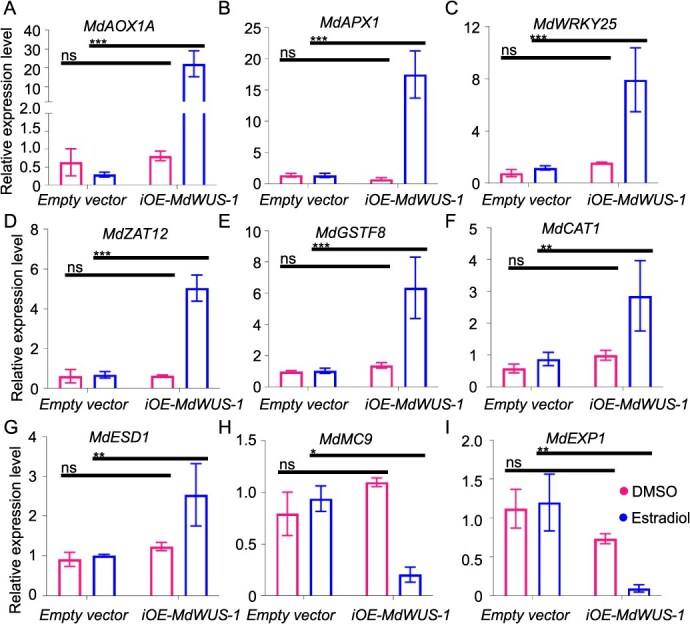
The expression level of oxidative stress, apoptosis, and cell growth-related genes of transgenic lines of *Malus* under the estradiol treatment. (A–I) The expression levels of *MdAOX1A*, *MdAPX1*, *MdWRKY25*, *MdZAT12*, *MdGSTF8*, *MdCAT1*, *MdESD1*, *MdMC9*, and *MdEXP1* genes in the empty vector control and *iOE-MdWUS-1* transgenic lines. Data are means ± SE (*n* = 3). Asterisks indicate significant differences by Student’s *t*-test (^*^P < 0.05).

This cell death appears to be triggered by oxidative stress or an overactive immune response. To explore this further, we analyzed the expression levels of several related genes, including *MdAOX1A*, *MdAPX1*, *MdWRKY25*, *MdZAT12*, *MdGSTF8*, *MdCAT1*, and *MdESD1*, the apoptosis-related gene *MdMC9,* and the cell growth-related gene *MdEXP1* (Fig. 7A–I). The results showed that, under estradiol-induced conditions, the expression of *MdAOX1A*, *MdAPX1*, *MdWRKY25*, *MdZAT12*, and *MdGSTF8* was significantly upregulated in the *iOE-MdWUS-1* lines, while *MdCAT1* and *MdESD1* showed only slight upregulation. In contrast, the expression of *MdMC9* and *MdEXP1* was significantly downregulated. These findings suggest that excessive expression of *MdWUS-1* induces oxidative stress and inhibits cell growth, ultimately leading to cell death.

## Discussion

We performed a phylogenetic analysis of the homeodomain superfamily, focusing on *WOX* subfamily, across 29 species. The phylogenetic tree revealed that ferns possess at least one *WUS* gene, with a protein length comparable to that of seed plants. We conducted pan-genome analysis of the *WOX* family in *Malus* and further examined the expression patterns of genes from modern branch of the *WOX* family in developing tissues and during regeneration processes in *Malus*. Through estradiol-inducible overexpression line of *MdWUS-1* and *MdWOX5-1*, we demonstrated that *MdWUS-1* plays a significant role in the LBS process, with its effect varying depending on the expression levels.

In the model plant *Arabidopsis thaliana*, the WUS protein consists of 292 amino acids. Its relatively small molecular weight allows WUS to diffuse from the OC to neighboring stem cells, a process essential for maintaining the SAM [8]. It has been shown that a modern branch *WOX* gene was first identified in *C*. *richardii*. This gene contains the same domains as the WUS protein but encodes a protein of 591 amino acids, which is much longer than AtWUS. The excessive molecular weight of this protein prevents it from diffusing between cells, resulting in loss of its function in maintaining the meristem. As a result, it was named the *CrWUL* gene [8, 25]. In 2022, the completion of several fern genome sequences facilitates the identification of a small group of fern genes in the *WOX* family classified into the modern subfamily. This group consists of six members (Fig. 1). Among them, we identified a fern gene from *Azolla filiculoides* (*Azfi_s0051.g031311*) that encodes a protein of only 303 amino acids, similar in length to AtWUS. This gene also contains a complete homeodomain, WUS–BOX domain, and EAR domain, and we named it *AfWUS* (Fig. 2A–C). This discovery indicates that the typical *WUS* gene existed in ferns long before the emergence of seed plants, offering new insights into the role of *WUS* in meristem development across plant lineages.

The current study demonstrates that moderate overexpression of *MdWUS-1* or *MdWOX5-1* significantly enhances the LBS in *Malus*, consistent with our earlier findings in RBS. Both *MdWUS-1* and *MdWOX5-1* promote shoot regeneration in both contexts, aligning with previous evidence showing that swapping the expression domains of *AtWUS* and *AtWOX5* in Arabidopsis still supports stem cell maintenance. This indicates that these genes are functionally similar, despite their distinct expression patterns in the shoot and root apices [8], underscoring the importance of promoter activity in determining the spatiotemporal regulation of these genes. Notably, *MdWUS-1* had a more pronounced effect on LBS, while *MdWOX5-1* was more effective in RBS, likely reflecting the evolutionary divergence in the gene ontologies of these homologous genes. Under native conditions, *AtWOX5* is expressed in the quiescent center of RAM, while *AtWUS* is localized to the SAM [3, 4]. This spatial specificity may explain why MdWUS-1 is more effective in LBS and *MdWOX5-1* in RBS, highlighting the evolutionary optimization of gene expression for efficient plant development.

Our results show that during plant growth and LBS regeneration, moderate overexpression of *MdWUS-1* significantly promotes growth, differentiation, and regeneration (Fig. 6L and M). However, excessively high levels of *MdWUS-1* gene expression result in an immediate cessation of growth and differentiation, ultimately leading to explant death. A similar phenomenon has been observed in maize, where overexpression of *ZmWUS2* causes callus browning and prevents bud regeneration [21]. Excessive *WUS* expression triggers the upregulation of oxidative stress-related genes and the downregulation of cell growth-related genes (Fig. 7). After injury, plants employ a bet-hedging strategy, balancing regeneration and defense [26]. In tomato, activation of local defense mechanism promotes regeneration, whereas systemic defense suppresses it [27]. This illustrates the close relationship between regeneration and defense. *AtWUS* has been shown to trigger innate antiviral immunity in plant stem cells by inhibiting protein synthesis [28]. Excessive inhibition of protein synthesis induces oxidative stress, and the high levels of *MdWUS-1* expression likely leads to severe suppression of protein synthesis, contributing to cellular stress and impaired regeneration. Several important questions remain for further investigation: What is the critical threshold of *MdWUS-1* expression and reactive oxygen species levels that determines the transition between promoting and inhibiting regeneration? Why does *MdWOX5-1* not exhibit a similar effect at present? Could this difference be attributed to gene evolution? *MdWUS-2* and *MdWOX5-2* are also of interest, as they may represent functional differentiation within this gene family and warrant further exploration.

## Materials and methods

### Identification and alignment of HOMEOBOX super family and *WOX* family

The candidate genes were accurately screened by searching for the conserved HB domain of the corresponding proteins. The public databases, including JGI Genome Portal (https://phytozome-next.jgi.doe.gov/), NCBI Conserved Domain Database https://www.ncbi.nlm.nih.gov/cdd), Plant Flowering-time Gene Database (https://doi.org/10.1093/hr/uhae013), plantGIR (https://doi.org/10.1093/hr/uhae342), Plant Hormone Gene Database (https://doi.org/10.1093/hr/uhae013) and Pfam database (http://pfam.xfam.org/) were used to search the HB domain of candidate sequences, and the domain ID is PF00046 in each database, respectively [29–34]. Sequences without the complete conserved HB domain were removed. The sequence information used is listed in Table S1.

Homologous protein sequences of *MdWOX* and *AtWOX* family genes were identified across 29 species using BLAST. The identified homologous sequences were aligned using MAFFT software with the parameter-m MFP, enabling the software to automatically test for the optimal model. Subsequently, a phylogenetic tree was constructed using IQ-TREE, with 1000 iterations set for ultrafast bootstrap analysis and SH-aLRT testing. These approaches ensured the reliability and robustness of the resulting phylogenetic tree. The sequence information used is listed in Table S2.

### Conserved protein motif analysis

This was performed using the MEME suite website (https://meme-suite.org/meme/tools/meme). The number of motifs to be discovered was set to 50, with a minimum motif width of 4 and a maximum motif width of 50.

### The pan genome analysis of *WOX* gene family of *Malus*

The GDR database (https://www.rosaceae.org) was used to obtain the genome data of the eight *Malus* varieties. The analysis of synteny, gene structure, and amino acid sequence homology was performed with TBtools [35]. Regulatory regions for each gene were extracted using a Perl script, defining the 3000 bp upstream of each gene. These regions were submitted to the PlantCARE database for cis-regulatory element prediction. To visualize the results, the data were organized and plotted using the TBtools, allowing a clear representation of the distribution and functional classification of the regulatory elements. The sequence information used is listed in Table S3.

### Generation of transgenic plants

Leaves from 30-day-old subcultured wild-type Gala seedlings were used as materials for genetic transformation. Transgenic apple plants were generated using the *Rhizobium rhizogenes*-mediated RBS transformation method. The procedures for vector construction, transformation steps, and expression level analysis were identical to those described in our previous study [36].

### Expression pattern analysis

To analyze the tissue-specific expression patterns of WUS gene family members, samples were collected from a 5-year-old apple tree grown in the field. RNA was isolated using the OmniPlant RNA Kit (CWBIO, Beijing, China). Single-stranded complementary DNA was synthesized using the Prime-Script™ RT reagent Kit with genomic DNA Eraser (Takara, Osaka, Japan). Quantitative RT-PCR was performed using the SYBR Green Pro Taq HS (Accurate, Taiwan, China) and a CFX96™ Real-Time PCR Detection System (Bio-Rad, Hercules, CA, USA). All primers used in this assay are listed in Table S5.

### Vibration sectioning and staining observation

A 2.5% paraformaldehyde solution was prepared in 1× PBS. Leaf and stem segments were immersed in the solution and fixed overnight at 4°C. The fixed samples were embedded in 5% low-melting-point agarose (purchased from Beyotime, China). Sections were performed at a thickness of 30 μm using a vibratome (Leica VT1200S, Leica Microsystems, Germany). After sectioning, the samples were stained overnight with Fluorescent Brightener 28 (Thermo Fisher Scientific, USA), rinsed with 1× PBS, and observed under the fluorescence microscope (Zeiss, Germany) with an excitation wavelength of 350 nm.

### LBS experiment

For the LBS experiment, mature leaves from subcultured plants grown for 90 days were used. Initially, 3–4 incisions were made on each leaf. The injured leaves were placed upside down on LBS medium. The LBS medium composition was 4.43 g/L MS salts, 2 mg/L TDZ, 0.5 mg/L NAA, 30 g/L sucrose, 7 g/L agar, and 300 mg/L timentin, adjusted to pH 5.6. The cultures were incubated in darkness at 25°C for 20 days, after which the leaves were transferred to fresh LBS medium and cultured under a photoperiod of 16 hours light/8 hours dark at 25°C for an additional month. For analyzing the expression levels of *MdWUS* gene family members during RBS, leaves from 30-day-old subcultured wild-type ‘Gala’ were used. The cultivation method was identical to the one described above.

### Statistical analysis

Data were analyzed using Student’s *t*-test (two-sided). Statistical significance was set at *P*-value <0.05.

## Supplementary Material

Web_Material_uhaf117

## Data Availability

Supplementary figures and supplementary data are available at Horticulture Research online.
