# Supplementary material for: The pan genome analysis of WOX gene family in apple and the two sides of MdWUS-1 in promoting leaf-borne shoot
Source: Hortic Res. 2025 Jul 11;12(8):uhaf117. doi: 10.1093/hr/uhaf117 (PMC12258036; doi:10.1093/hr/uhaf117)

Figure S1

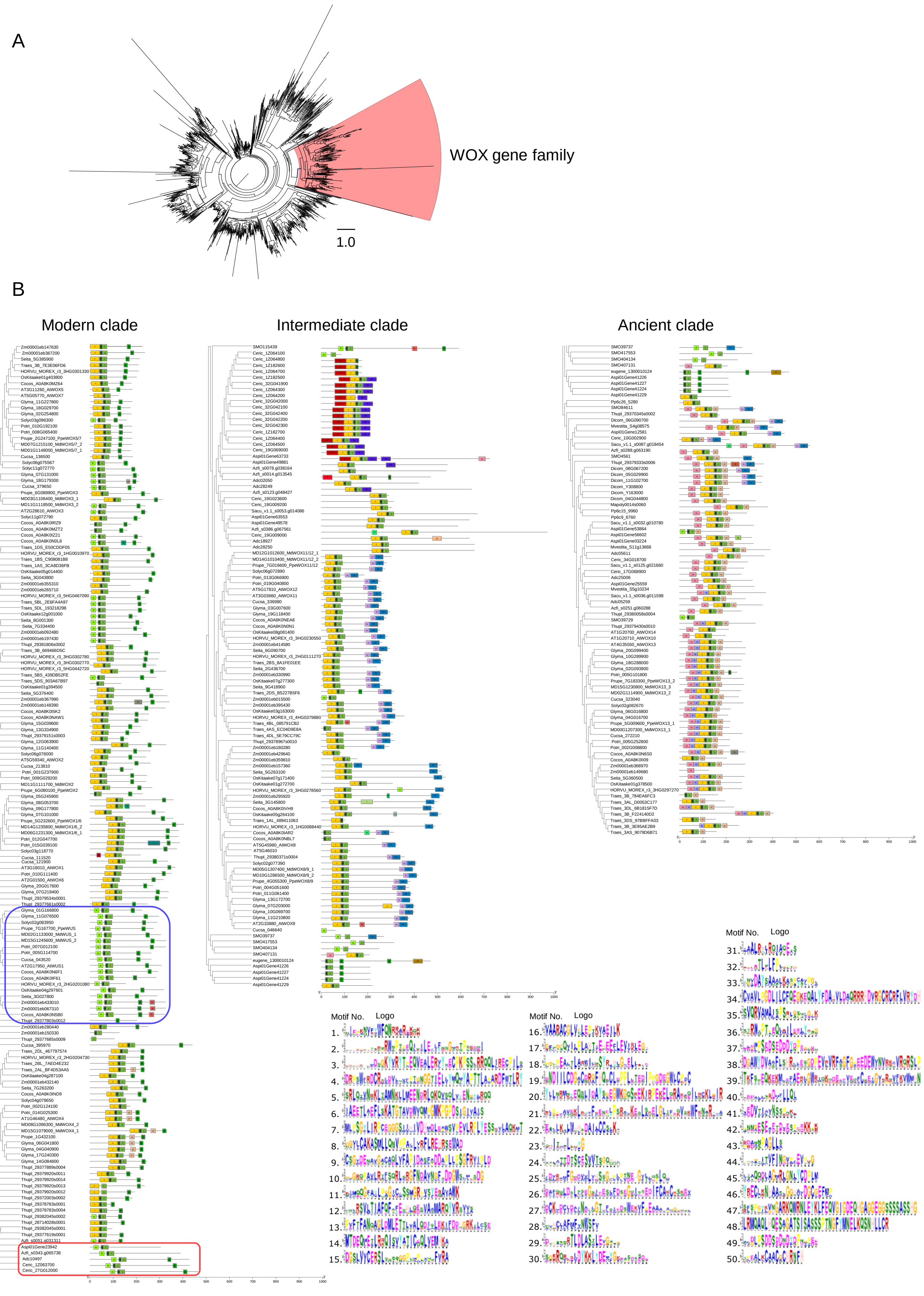
A
WOX gene family
1.0
B
Modern clade
Intermediate clade
Ancient clade
Motif No.
Logo

Figure S2

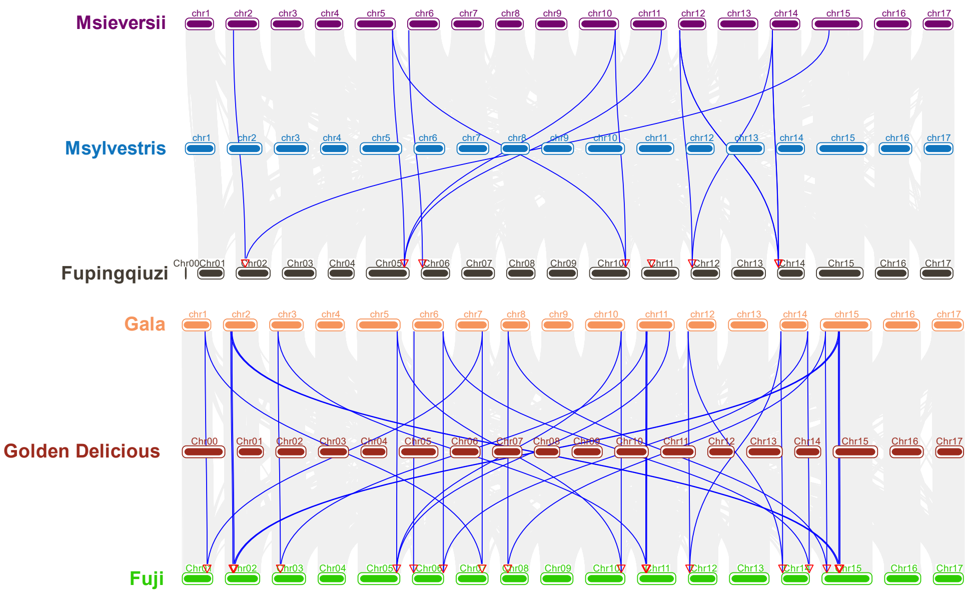
Msieversii
Msylvestris
Fupingqiuzi
Gala
Golden Delicious
Fuji

Figure S3

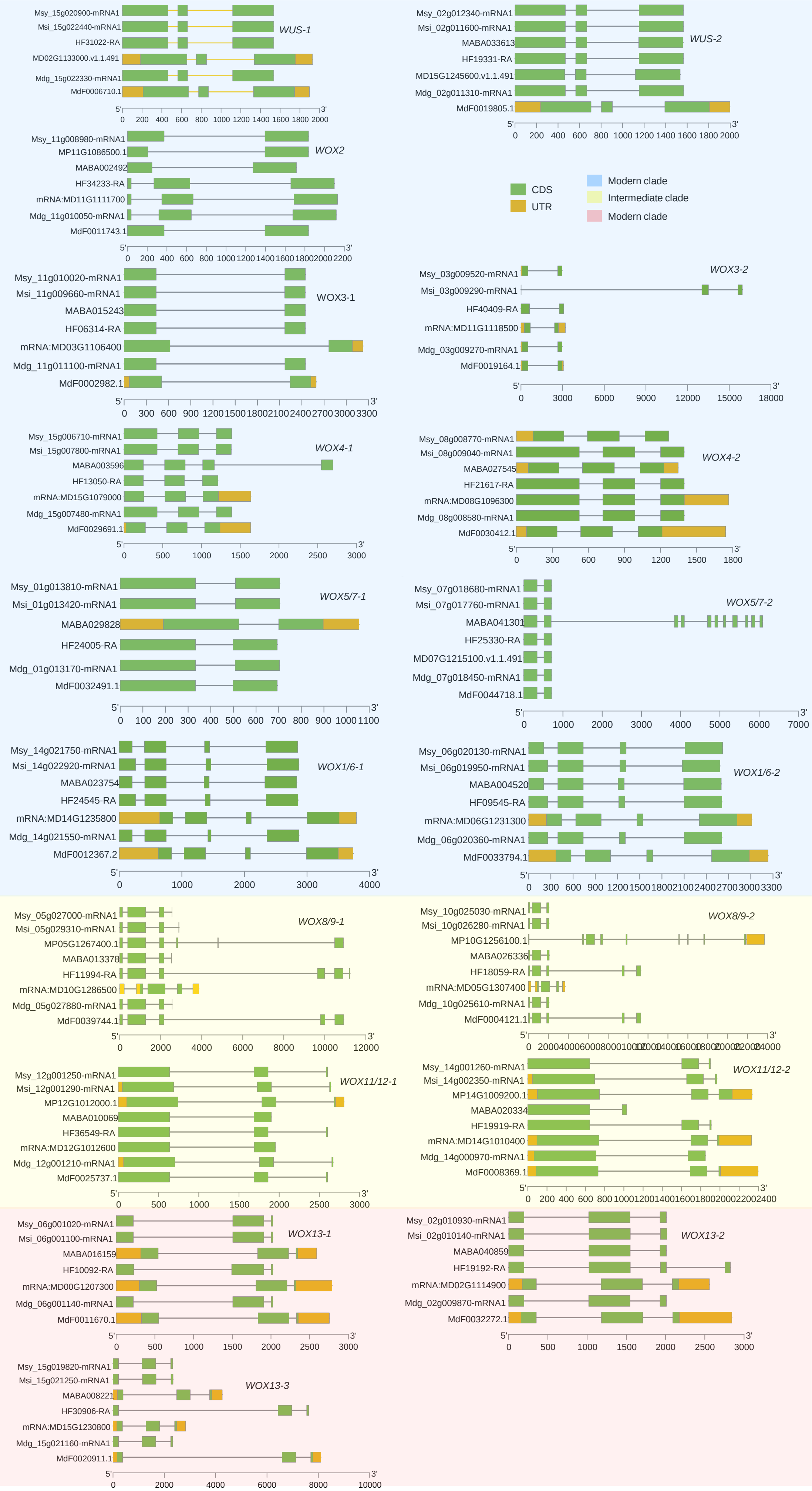

Msy_15g020900-mRNA1
Msi_15g022440-mRNA1
HF31022-RA
MD02G1133000.v1.1.491
Mdg_15g022330-mRNA1
MdF0006710.1
WUS-1
Msy_02g012340-mRNA1
Msi_02g011600-mRNA1
MABA033613
HF19331-RA
MD15G1245600.v1.1.491
Mdg_02g011310-mRNA1
MdF0019805.1
WUS-2
Msy_11g008980-mRNA1
MP11G1086500.1
MABA002492
HF34233-RA
mRNA:MD11G1111700
Mdg_11g010050-mRNA1
MdF0011743.1
WOX2
CDS
UTR
Modern clade
Intermediate clade
Modern clade
Msy_11g010020-mRNA1
Msi_11g009660-mRNA1
MABA015243
HF06314-RA
mRNA:MD03G1106400
Mdg_11g011100-mRNA1
MdF0002982.1
WOX3-1
Msy_03g009520-mRNA1
Msi_03g009290-mRNA1
HF40409-RA
mRNA:MD11G1118500
Mdg_03g009270-mRNA1
MdF0019164.1
WOX3-2
Msy_15g006710-mRNA1
Msi_15g007800-mRNA1
MABA003596
HF13050-RA
mRNA:MD15G1079000
Mdg_15g007480-mRNA1
MdF0029691.1
WOX4-1
Msy_08g008770-mRNA1
Msi_08g009040-mRNA1
MABA027545
HF21617-RA
mRNA:MD08G1096300
Mdg_08g008580-mRNA1
MdF0030412.1
WOX4-2
Msy_01g013810-mRNA1
Msi_01g013420-mRNA1
MABA029828
HF24005-RA
Mdg_01g013170-mRNA1
MdF0032491.1
WOX5/7-1
Msy_07g018680-mRNA1
Msi_07g017760-mRNA1
MABA041301
HF25330-RA
MD07G1215100.v1.1.491
Mdg_07g018450-mRNA1
MdF0044718.1
WOX5/7-2
Msy_14g021750-mRNA1
Msi_14g022920-mRNA1
MABA023754
HF24545-RA
mRNA:MD14G1235800
Mdg_14g021550-mRNA1
MdF0012367.2
WOX1/6-1
Msy_06g020130-mRNA1
Msi_06g019950-mRNA1
MABA004520
HF09545-RA
mRNA:MD06G1231300
Mdg_06g020360-mRNA1
MdF0033794.1
WOX1/6-2
Msy_05g027000-mRNA1
Msi_05g029310-mRNA1
MP05G1267400.1
MABA013378
HF11994-RA
mRNA:MD10G1286500
Mdg_05g027880-mRNA1
MdF0039744.1
WOX8/9-1
Msy_10g025030-mRNA1
Msi_10g026280-mRNA1
MP10G1256100.1
MABA026336
HF18059-RA
mRNA:MD05G1307400
Mdg_10g025610-mRNA1
MdF0004121.1
WOX8/9-2
Msy_12g001250-mRNA1
Msi_12g001290-mRNA1
MP12G1012000.1
MABA010069
HF36549-RA
mRNA:MD12G1012600
Mdg_12g001210-mRNA1
MdF0025737.1
WOX11/12-1
Msy_14g001260-mRNA1
Msi_14g002350-mRNA1
MP14G1009200.1
MABA020334
HF19919-RA
mRNA:MD14G1010400
Mdg_14g000970-mRNA1
MdF0008369.1
WOX11/12-2
Msy_06g001020-mRNA1
Msi_06g001100-mRNA1
MABA016159
HF10092-RA
mRNA:MD00G1207300
Mdg_06g001140-mRNA1
MdF0011670.1
WOX13-1
Msy_02g010930-mRNA1
Msi_02g010140-mRNA1
MABA040859
HF19192-RA
mRNA:MD02G1114900
Mdg_02g009870-mRNA1
MdF0032272.1
WOX13-2
Msy_15g019820-mRNA1
Msi_15g021250-mRNA1
MABA008221
HF30906-RA
mRNA:MD15G1230800
Mdg_15g021160-mRNA1
MdF0020911.1
WOX13-3

Figure S4

A

WUS-1 WUS-2

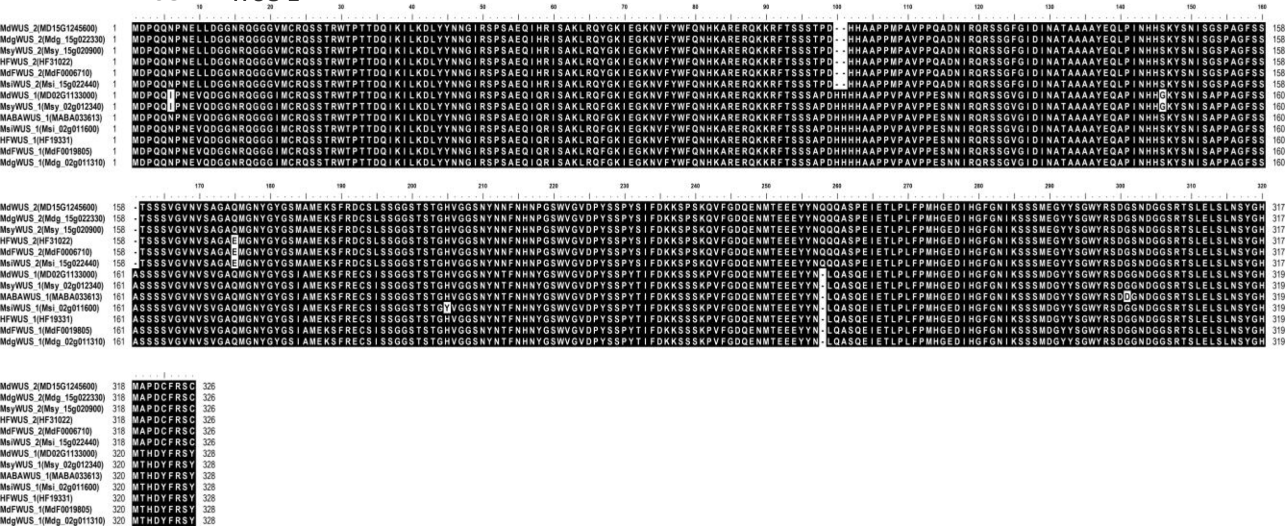

B

WOX2

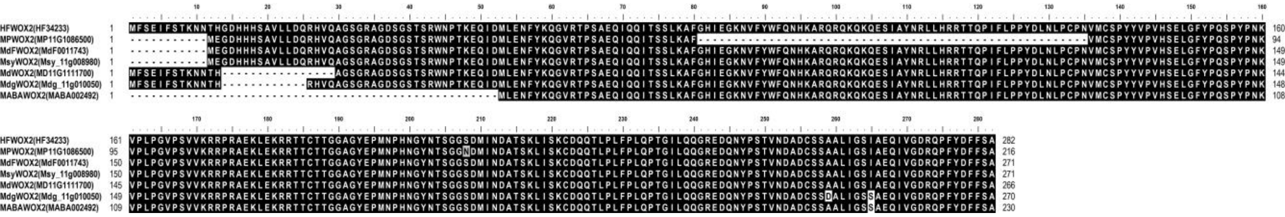

C

WOX3-1 WOX3-2

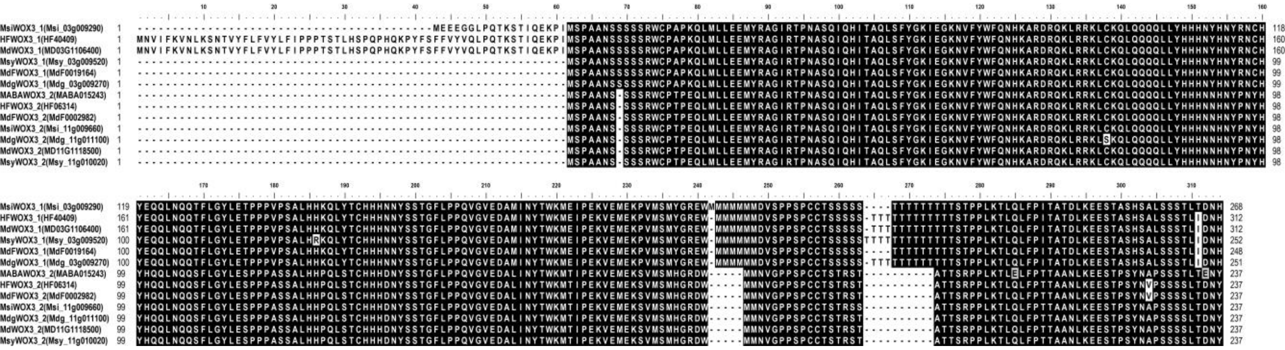

D

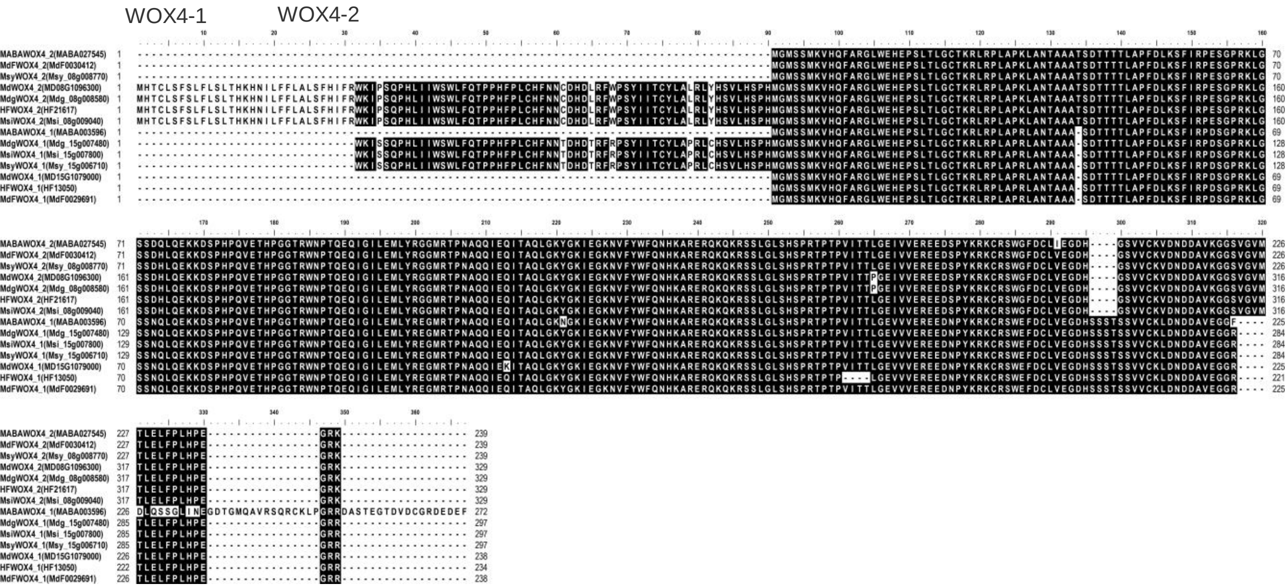

E

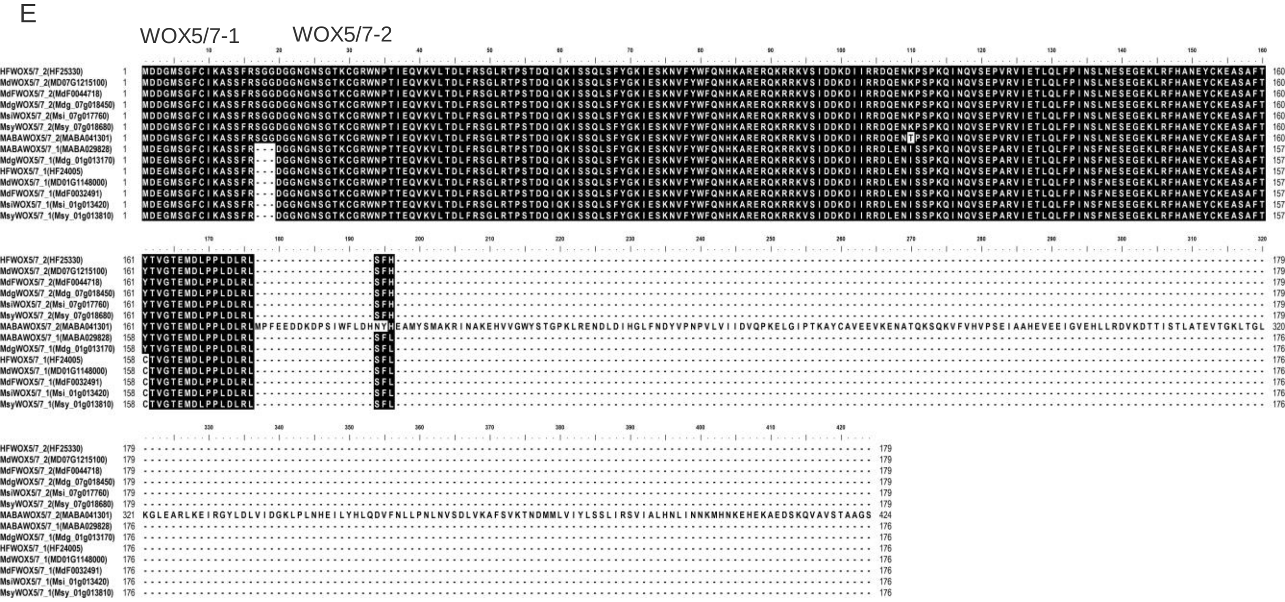

F

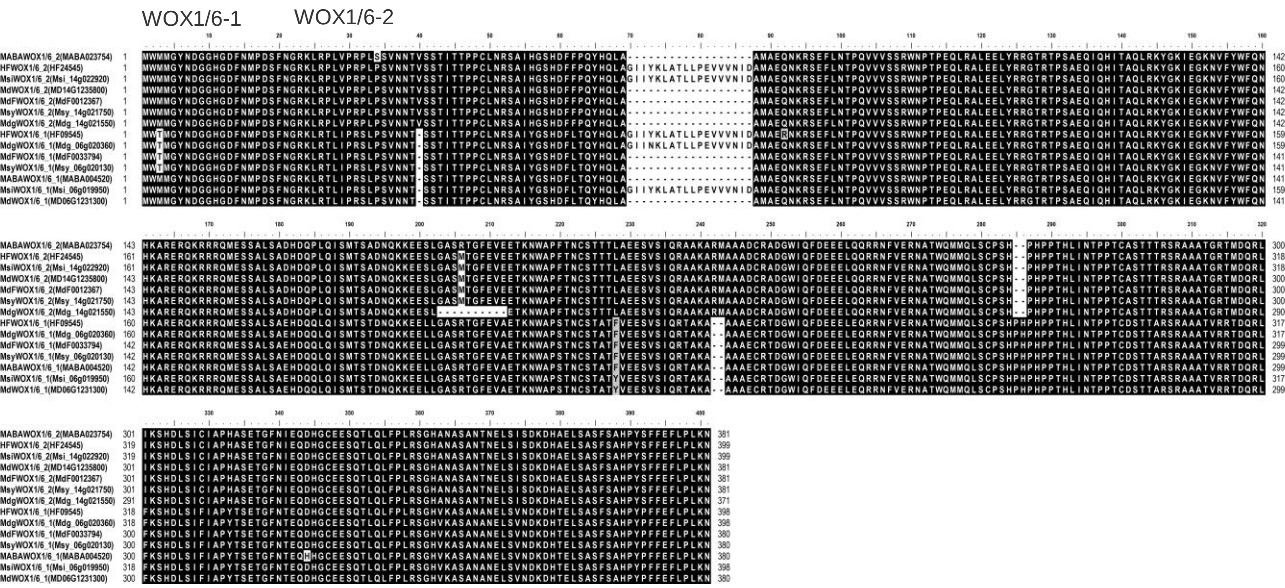

G

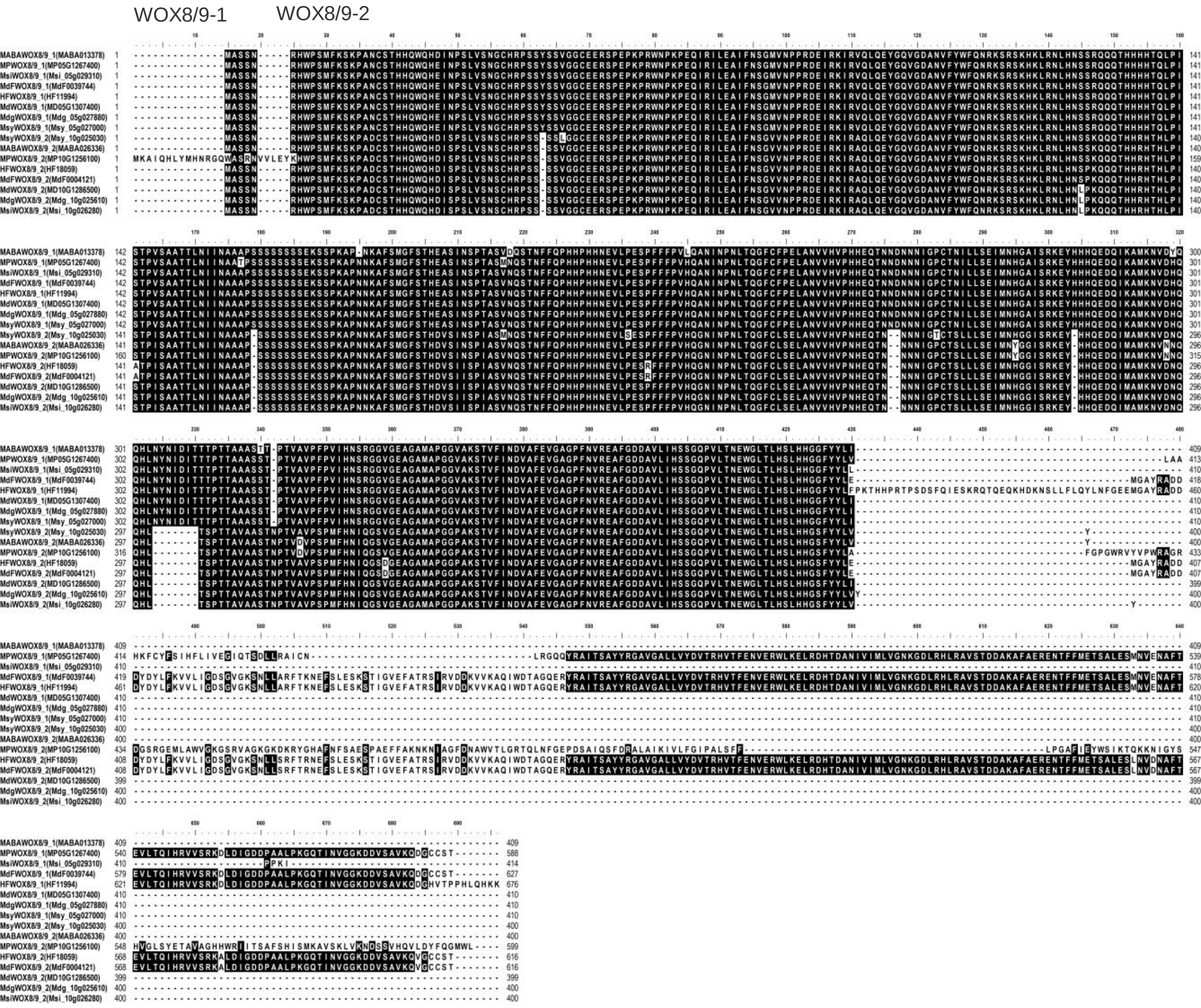

H

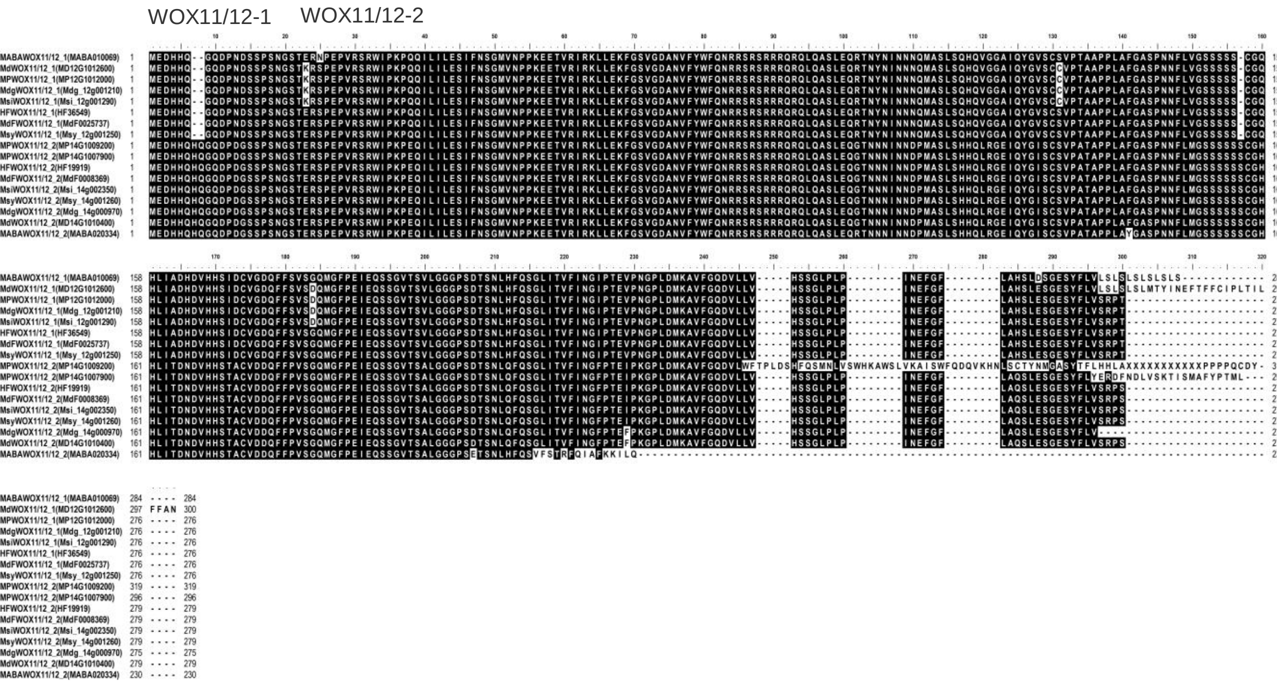

I

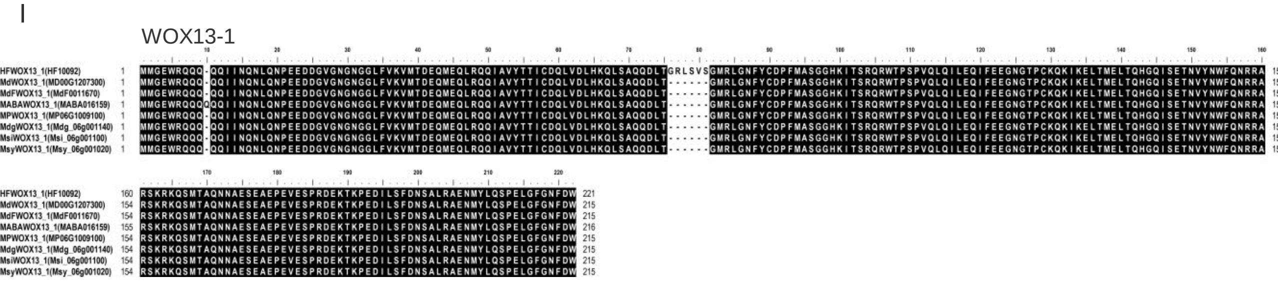

J

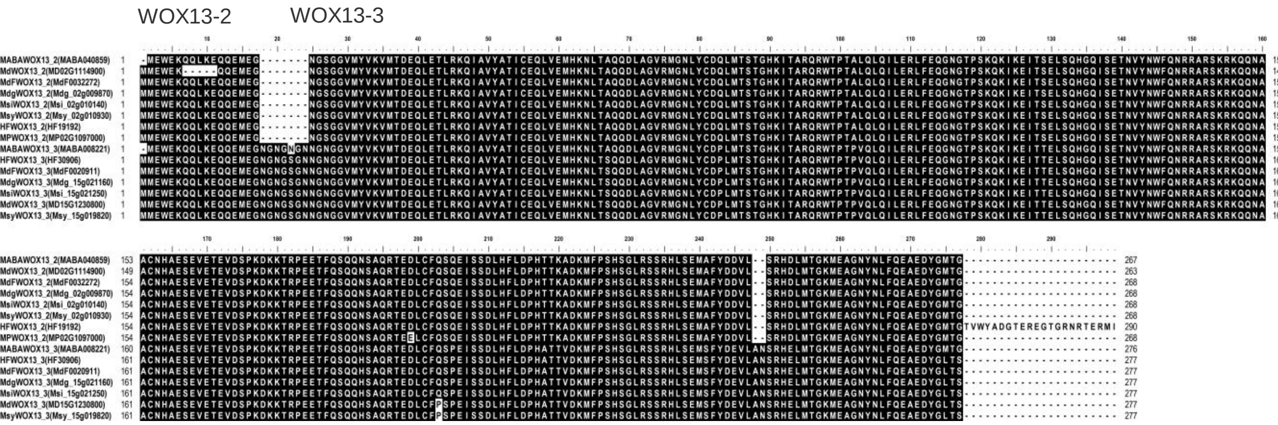

Figure S5

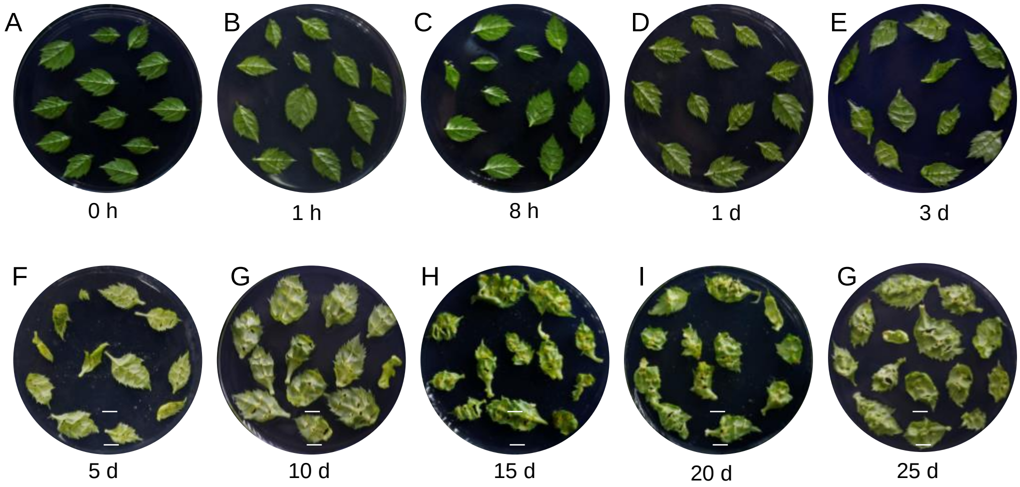

Figure S6

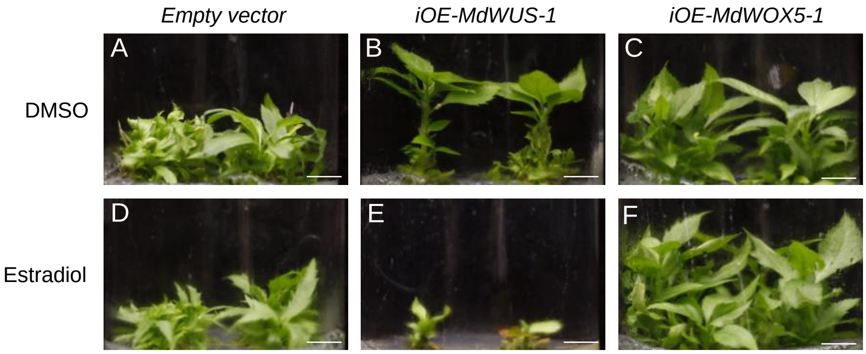

Figure S7

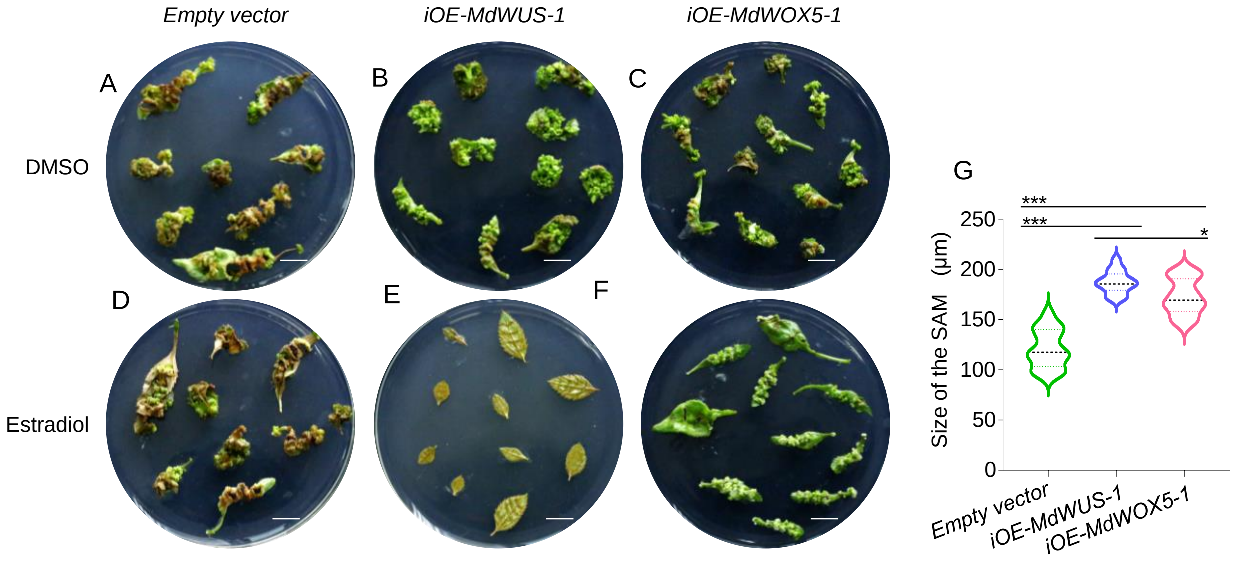

Supplement: Web_Material_uhaf117 [file web_material_uhaf117.zip › Supplementary figures.pdf]
